# Non-small cell lung cancer after EGFR-TKI resistance: from drug resistance mechanisms to precision interventions

**DOI:** 10.3389/fphar.2026.1795614

**Published:** 2026-04-22

**Authors:** Zhen Wang, Yanqi Song, Aidi Wang, Baoshan Liu

**Affiliations:** 1 Tianjin Medical University General Hospital, Tianjin, China; 2 Graduate School, Tianjin University of Traditional Chinese Medicine, Tianjin, China

**Keywords:** EGFR-TKIs, novel therapies, NSCLC, precision interventions, resistance mechanisms, therapeutic strategy

## Abstract

The emergence of epithelial growth factor receptor tyrosine kinase inhibitors (EGFR-TKIs) propelled EGFR-mutated patients of non-small cell lung cancer (NSCLC) into the era of precision medicine. Third-generation targeted therapies, such as osimertinib, can specifically target the T790M mutation and effectively overcome resistance to previous treatments, significantly prolonging progression-free survival in patients. Despite its remarkable clinical efficacy and manageable safety profile, it is still not immune to the development of resistance. Therefore, overcoming resistance and providing new treatment strategies for advanced NSCLC patients harboring EGFR mutations after osimertinib resistance are priorities that need to be considered. New-generation EGFR-TKIs or combination therapeutic strategies hold promise to address resistance. In addition, with the development of genomics and molecular diagnostic technologies, several drugs with novel mechanisms of action, such as antibody-drug conjugates and bispecific antibodies, have shown promising clinical response rates and favorable safety profiles in several clinical and experimental studies. This review aims to more systematically indicate the mechanisms of EGFR-TKI resistance and summarize the new strategies available and novel drugs under investigation for NSCLC patients harboring EGFR mutations after TKI resistance to extend their survival and improve quality of life.

## 1 Introduction

Lung cancer is currently the second-highest incidence and the highest mortality among malignant tumors worldwide (Bray et al., 2024). Non-small cell lung cancer (NSCLC) is the most common pathological type, accounting for roughly 85% of lung cancer (Bray et al., 2024). Epithelial growth factor receptor (EGFR) mutations are common in lung adenocarcinomas, the most frequent being the exon 19 and exon 21 L858R mutations, accounting for approximately 80%–90% of all mutations (Fu et al., 2022). Exon 20 insertion mutations are another rare type (Borgeaud et al., 2024). Exon 20 T790M mutations are mostly associated with tyrosine kinase inhibitor (TKI) resistance (Mok et al., 2017). The EGFR belongs to the human epidermal growth factor receptor (HER) family, also known as HER1, and is a group of cell surface receptors with tyrosine kinase activity (Sabbah et al., 2020). When a ligand binds to the extracellular domain of EGFR, it leads to the dimerization of the receptor itself with other members of the HER family, prompting adenosine triphosphate-mediated phosphorylation, which leads to the activation of downstream signaling pathways implicated in proliferation, apoptosis, and proliferation of cells, etc (Abourehab et al., 2021; Levantini et al., 2022).

According to data, the proportion of EGFR mutations in Asian lung adenocarcinoma patients is significantly higher than in Western countries (Graham et al., 2018). Generally, patients who undergo genetic testing that shows EGFR mutations have a high efficacy in TKIs (Maemondo et al., 2010). However, the majority of patients experienced disease progression at a median of 12 months of treatment with first- or second-generation TKIs, which was mostly associated with resistance due to T790M mutations (Remon et al., 2014).

To address this problem, the third-generation EGFR-TKI, osimertinib, was introduced and approved for treating NSCLC patients with EGFR and T790M mutations. Unfortunately, during osimertinib treatment, some patients inevitably develop acquired resistance, leading to disease progression (He J. et al., 2021). The exact mechanisms of osimertinib resistance remain unclear and may be connected to one or several mechanisms, as well as heterogeneity among resistance mechanisms (Zhao et al., 2022).

The resistance mechanisms of osimertinib are multifactorial and not fully understood. Most studies on the resistance mechanism of osimertinib have focused on acquired resistance until now (Zalaquett et al., 2023). Primary resistance is mostly related to the exon 20 insertion mutation (Shah and Neal, 2022). Targeted therapy is the preferred option for NSCLC patients with EGFR mutations, but what to do for patients after TKI therapy resistance remains a critical consideration. A new generation of EGFR-TKIs is being investigated to overcome the deficits of third-generation TKIs. Also, novel drugs such as monoclonal antibodies, bispecific antibodies, and ADCs are being researched in parallel. In addition, combination therapeutic strategies are also an optional direction for advanced NSCLC patients with EGFR-mutated and TKI resistance. This review multifacetedly summarizes the resistance mechanisms to three generations of TKIs and generalizes new therapeutic strategies and novel agents in the investigation to improve clinical outcomes for patients.

## 2 Acquired resistance mechanisms

### 2.1 On-target resistance mechanisms

Gefitinib and erlotinib, first-generation TKIs, are reversible inhibitors, and second-generation TKIs (represented by afatinib) are irreversible inhibitors that covalently bind to the EGFR. Several clinical trials have demonstrated that single-agent gefitinib or afatinib provides greater survival benefits and significantly prolongs progression-free survival (PFS) of patients compared to conventional chemotherapy (Johnson et al., 2022), such as NEJ002, IPASS, LUX-Lung 3, CONVINCE, WJTOG3405, and ARCHER 1050 studies (Fukuoka et al., 2011; Inoue et al., 2013; Mok et al., 2021; Sequist et al., 2023; Yoshioka et al., 2019). Studies have also validated the decision between first or second-generation inhibitors, with second-generation agents being more sensitive to EGFR mutations and less prone to resistance than first-generation TKIs (Yu et al., 2013). Simultaneously, the choice of second-generation TKIs provides superior PFS. However, side effects associated with therapy have also increased. Thus, the decision must be made by expert clinicians. The studies have found that the EGFR T790M mutation was the main cause of resistance to first and second-generation TKIs, as well as the main source of resistance after initial relief in patients with lung cancer receiving treatment, which is associated with the EGFR exon 20 insertions (Meador et al., 2021). T790M, which results from the substitution of methionine for threonine at amino acid 790 in exon 20 of the EGFR, stops TKIs from binding to the adenosine triphosphate (ATP)-binding site of the receptor and reduces the downstream signaling pathway of the EGFR, which ultimately leads to the occurrence of resistance (Laface et al., 2023).

To address the resistance phenomenon, researchers have accelerated their research and development of third-generation TKIs, particularly the marketing of osimertinib, which has dramatically improved the resistance problem induced by the T790M mutation. It is noteworthy that long before the development of osimertinib, Zhou et al. (Zhou et al., 2009) had already creatively proposed the first third-generation EGFR-TKI based on a pyrimidine skeleton, aiming to pioneer a covalent inhibitor targeting the T790M mutation. Third-generation EGFR-TKIs structurally abandon the quinazoline skeleton of first- and second-generation inhibitors, replacing it with an aminopyrimidine core to achieve highly selective inhibition of L858R/T790M mutations. This provided the core structure and mechanism basis for the design of third-generation TKIs, such as osimertinib.

In November 2015, the Food and Drug Administration (FDA) first approved osimertinib, a representative drug of the third-generation TKIs, for the treatment of patients with NSCLC who have been or are being treated with EGFR-TKIs and have a T790M mutation, and since then, its status has even improved from second-line to first-line therapy (Soria et al., 2018). Osimertinib also shows potential efficacy in patients with EGFR mutations who have developed central nervous system (CNS) metastases due to its ability to penetrate the blood-brain barrier (Janne et al., 2024). Multiple studies have indicated the superior clinical efficacy and safety of osimertinib versus gefitinib or platinum-containing chemotherapy, with patients experiencing significantly longer PFS and overall survival (OS) and lower incidence of adverse events (AEs) (Mok et al., 2017; Lu et al., 2022a). Nonetheless, there is still a portion of patients who develop resistance after treatment with osimertinib, which accounts for 10%–20% of patients, and the mechanisms in the majority of patients are still unclear (Girard, 2022). The C797S in exon 20 of EGFR is the leading mechanism for treatment resistance to osimertinib when administered as a backline therapy (Zhao et al., 2022; Li Y. et al., 2023; Mu et al., 2020).

**FIGURE 1:**
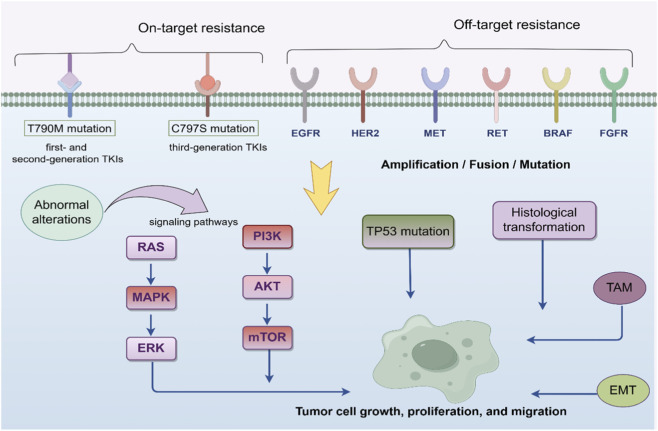
The potential mechanisms of resistance to EGFR-TKIs^a^. a TKIs, tyrosine kinase inhibitors; EGFR, epithelial growth factor receptor; HER2, human epidermal growth factor receptor 2; MET, mesenchymal to epithelial transition factor; RET, rearranged during transfection; BRAF, V-RAF murine sarcoma viral oncogene homolog B1; FGFR, fibroblast growth factor receptor; RAS/MAPK/ERK, Ras/mitogen-activated protein kinase/Extracellular regulated protein kinases; PI3K/AKT/mTOR, Phosphatidylinositol-3-kinase/Akt/Mammalian target of the rapamycin; TAM, tumor-associated macrophages; EMT, epithelial-mesenchymal transition; TP53, tumor protein p53.

The C797S mutation reduces the sensitivity of osimertinib to the C797 residue and instead increases the ability to bind with ATP, which renders a significant reduction of the inhibitory action of osimertinib towards tumor cells (Diaz-Serrano et al., 2018). It has been found that EGFR C797G and C797N, L718Q, EGFR Leu718/Gly719, L844V, and G724 mutations are also engaged in resistance to third-generation EGFR-TKIs (Le et al., 2018; Peled et al., 2017; Yang et al., 2018; Zhao et al., 2020). The potential mechanisms of resistance to osimertinib are shown in Figure 1.

### 2.2 Off-target resistance mechanisms

#### 2.2.1 Oncogene amplification, fusion

HER2 amplification and mesenchymal to epithelial transition factor (MET) amplification are the main mechanisms of resistance to EGFR-TKIs (Mu et al., 2020; Schoenfeld and Yu, 2020). MET is a receptor tyrosine kinase that regulates biological processes such as cell proliferation, apoptosis, and migration. MET is abnormally expressed or gene-amplified in a variety of malignant tumors, and the signaling pathways of MET-mediated play significant roles in the occurrence and development of cancers (Remon et al., 2023). HER2, a proto-oncogene, is mutated or amplified in lung, breast, and gastric cancer, and is engaged in the process of inhibiting tumor cell apoptosis, promoting cancer cell proliferation, and invasion (Vathiotis et al., 2021). Previous studies have concluded that MET amplification is correlated with resistance to first or second-generation TKIs. At present, it has been confirmed that MET amplification is closely related to third-generation TKI resistance (Nishiyama et al., 2020). HER2 directly activates EGFR downstream signaling to exert tumor-promoting functions and mediate TKI resistance. In addition, it has been found that HER2 exon 20 insertion is also associated with resistance to osimertinib (Proto et al., 2017; Ramalingam et al., 2018). Oncogenic gene fusions are another reason for resistance to third-generation TKIs. Some studies have found that the fusion of oncogenes such as Rearranged during transfection (RET), V-RAF murine sarcoma viral oncogene homolog B1 (BRAF), anaplastic lymphoma kinase (ALK), and fibroblast growth factor receptor (FGFR) is highly associated with osimertinib resistance (Aboubakar and Ocak, 2021; Piotrowska et al., 2018; Solassol et al., 2019; Zeng et al., 2021). Currently, this type of resistance is expected to be addressed by combining EGFR TKIs with MET inhibitors, RET inhibitors, and BRAF inhibitors.

#### 2.2.2 Histological transformation

NSCLC, especially adenocarcinoma transformed into small cell lung cancer (SCLC), is one of the causes of TKI resistance in patients with EGFR mutations. A retrospective clinical study found that 0.7% of patients (7/1012) were diagnosed with SCLC at the time of biopsy (Chen et al., 2023a), whereas these patients had EGFR-sensitive lung adenocarcinoma at the time of diagnosis. Next-generation sequencing showed that the phenotypic transformation may be associated with Tumor protein p53 (TP53) gene mutations and deletion of the RB1 gene. Furthermore, the study revealed that patients with phenotypically transformed EGFR mutations had worse responses to treatment and poor clinical outcomes. Nicolas Marcoux et al. discovered that patients with EGFR-mutated SCLC responded badly to chemotherapy and had significantly shorter survival than patients with EGFR-mutated NSCLC (Marcoux et al., 2019). This histological transformation can also be converted from adenocarcinoma to squamous cell carcinoma, and the survival time of patients is shortened (Schoen et al., 2020).

#### 2.2.3 Abnormal alterations in signaling pathways

There are numerous signaling pathways downstream of EGFR, such as Janus kinase/signal transducer and activator of transcription (JAK/STAT), mitogen-activated protein kinase/Ras/Extracellular regulated protein kinases (RAS/MAPK/ERK), and Phosphatidylinositol-3-kinase/Akt/Mammalian target of the rapamycin (PI3K/AKT/mTOR), and abnormal alterations in these pathways can mediate the resistance of osimertinib (Rosell et al., 2021; Wu and Lin, 2022). A study found that the changes in PI3K/AKT/mTOR signaling pathway-related genomics were detected in patients with TKI resistance (Fuchs et al., 2021; Lee et al., 2021; Liu et al., 2024). The mutation is prevalent in solid tumors, including breast, small-cell lung, and endometrial cancers (Lengel et al., 2023). PIK 3CA mutations can also be observed in some patients with NSCLC (5%–8%) (Lengel et al., 2023). This mutation can activate the PI3K/AKT signaling pathway, promote tumor cell survival and proliferation, and decrease the efficacy of TKIs, which leads to TKI resistance (He Y. et al., 2021; Jee et al., 2022). Currently, PI3K inhibitors for solid tumors with PIK3CA mutations are under investigation (Belli et al., 2023; Krop et al., 2022).

#### 2.2.4 Other mechanisms

Epithelial-mesenchymal transition (EMT) may also be involved in TKI resistance. It has been demonstrated that EMT dysfunction is closely associated with the occurrence and development of lung cancer, and may be involved in the process of cell proliferation, apoptosis, and invasion as well as resistance (Jiang XM. et al., 2021). The researchers found that EMT was detectable in patients with lung cancer with EGFR mutations, which may be associated with disease progression (Singh and Settleman, 2010; Weng et al., 2019).

TP53 mutations are generally found in a variety of malignant tumors, with lung cancer being the most frequently affected (Wang et al., 2022). The P53 protein, which is crucial to the cell cycle, is encoded by TP53. TP53 mutation is also an essential driver of tumor development and drug resistance. TP53 mutations have been shown to contribute to resistance to EGFR-TKIs and reduce PFS (Chang et al., 2020).

APR-246, also known as PRIMA-1MET, is a small-molecule compound capable of restoring the normal conformation and anti-tumor transcriptional activity of mutant p53 proteins, enabling cells to reactivate the expression of p53 target genes, which in turn impacts cell cycle regulation and apoptosis induction to exert anti-cancer effects (Michaeli et al., 2024). Likewise, PRIMA-1 is a small molecule that, together with its main active metabolite 2-methyl-3-quinolone, restores mutant p53 to a conformation similar to that of the wild type, thus reactivating the transcriptional activity of p53 and inducing apoptosis. It is well-tolerated and has preliminary antitumor activity in relevant trials. MK-1775 (Adavosertib) is a Wee1 kinase inhibitor. TP53-mutated tumor cells rely on the G2-phase checkpoint to maintain genomic stability, whereas MK-1775 disrupts the G2-phase checkpoint through inhibition of the Wee1 kinase, resulting in the inability of these cells to normally repair DNA damage, thereby triggering apoptosis. At present, several clinical trials, including APR-246, MK-1775, and PRIMA-1, are ongoing, which may offer new hope to patients of NSCLC with TP53 mutation (Fujihara et al., 2022; Kong and Mehanna, 2021; Wang et al., 2023).

It was found that tumor-associated macrophages (TAM) are also engaged in mediating EGFR-TKI resistance (Cheng et al., 2023). TAM mediates TKI resistance by stimulating signaling pathways, inhibiting T cell-mediated responses, inducing macrophage polarization to M2 type, and regulating the phenotype of tumor cells. Currently, therapeutic strategies aimed at TAM are in progress, including inhibition of downstream signaling pathway activation, reprogramming of macrophage phenotype, and induction of macrophage polarization to M1 type, which may reverse TKI inhibition of drug resistance and enhance its anticancer effects (Liang et al., 2022; Wang et al., 2020; Yu et al., 2022; Zheng et al., 2021).

Cell-protective autophagy plays an important role in EGFR-TKI resistance. Glycosylated lysosomal membrane protein (GLMP) is a transmembrane glycoprotein. Some researchers have found that it is abnormally expressed in drug-resistant cells (Liang et al., 2025). Researchers collected clinical lung cancer tissue samples before and after resistance to EGFR-TKI therapy. They found that both mRNA and protein levels of GLMP were remarkably higher in tumor tissues after resistance developed versus before treatment. In the constructed TKI-resistant cell lines, GLMP expression was also found to be significantly upregulated. During mechanism analysis, they discovered that GLMP confers TKI resistance to tumor cells by promoting protective autophagy. Additionally, studies revealed that GLMP activates the Ras Homologous Gene Family Protein A (RhoA)/Rho-associated coiled-coil forming protein kinase signaling pathway, and activation of the RhoA pathway is typically associated with EMT in tumor cells. EMT is one of the key mechanisms underlying TKI resistance. The results indicate that GLMP is likely to become a new target for overcoming TKI resistance.

A study has explored the mechanism of Ambra1 in TKI resistance in non-small-cell lung cancer (Chen et al., 2023b). Ambra1 is a pivotal protein in the autophagy initiation process, mainly promoting autophagosome formation by binding to and stabilizing Beclin1. In the constructed gefitinib-resistant cell strains, both mRNA and protein levels of Ambra1 were detected to be notably higher than those in the parental sensitive cells. This phenomenon is also found in clinical samples. The emergence of Ambra1-mediated drug resistance is closely related to its induction of protective autophagy. Researchers further investigated why Ambra1 is highly expressed in resistance cells. They observed that this may be implicated in the activation of the STAT3 signaling pathway. Moreover, treatment with autophagy inhibitors in conjunction with TKI can reverse resistance.

In addition, deletion of T790M, amplification of fibroblast growth factor (FGF)/FGFR, and abnormal activation of insulin-like growth factor 1 receptor (IGF1R) may be potential mechanisms of EGFR-TKI resistance (Bertoli et al., 2022; Makimoto et al., 2021; Manabe et al., 2020; Murtuza et al., 2019). Table 1 shows the approved third-generation EGFR-TKIs. Table 2 shows several ongoing trials for third-generation EGFR-TKIs.

**TABLE 1:** Summary of approved third-generation EGFR-TKIs^a^.

Drug name	Clinical trial	Primary outcomes	Comparison	Latest approval date
Osimertinib	FLAURA2	mPFS: 25.5 vs. 16.7 months	Osimertinib plus chemotherapy vs. osimertinib	February 2024
Almonertinib	AENEAS	CNS mPFS: 29 vs. 8.3 months	Almonertinib vs. gefitinib	December 2021
Furmonertinib	FURLONG	mPFS: 20.8 vs. 11.1 months	Furmonertinib vs. gefitinib	June 2022
Befotertinib (D-0316)	IBIO-103	mPFS: 22.1 vs. 13.8 months	Icotinib vs. D-0316	May 2023
Rilertinib	SHC013-II-01	DCR: 55.9% and 60.4% mPFS: 12.4 and 12.6 months mOS: 26.0 months and NR	Rilertinib Part A and part B	June 2024
Rezivertinib	NCT03812809	mPFS: 12.2 months mOS: 23.9 months CNS mPFS: 16.6 months	Rezivertinib (BPI-7711)	May 2024
Lazertinib	MARIPOSA	mPFS: 23.7 vs. 16.6 months mDOR: 25.8 vs. 16.7 months	Lazertinib plus amivantamab vs. osimertinib	August 2024
Sunvozertinib	WU-KONG6	ORR: 60.8% DCR: 87.6%	Sunvozertinib	August 2023

^a^ EGFR-TKIs, epithelial growth factor receptor tyrosine kinase inhibitors; mPFS, median progression-free survival; CNS, central nervous system; DCR, disease control rate; mOS, median overall survival; mDOR, median duration of response; ORR, objective response rate.

**TABLE 2:** Several ongoing trials for third-generation EGFR-TKIs^a^.

Drug name	NCT number	Phase	Enrollment	Primary end points	Secondary end points
YK-029A	NCT05767892	Phase 3	350	PFS	ORR, OS, DCR, etc.
Limertinib (ASK120067)	NCT04143607	Phase 3	337	PFS	DOR, DCR, ORR, OS
D-0316	NCT04206072	Phase 2/3	362	PFS	DCR, ORR, AEs, etc.
Rezivertinib (BPI-7711)	NCT03812809	Phase 2b	226	ORR	ORR, PFS, OS
BEBT-109	NCT06514027	Phase 2	30	ORR, AEs	DCR
FHND9041	NCT06521034	Phase 1/2	124	Cmax, AUC	ORR, AEs, PFS
Abivertinib (AC0010)	NCT02330367	Phase 1/2	368	RP2D, ORR	MTD, DLT, PFS, etc.
ES-072	CTR20180074	Phase 1	19	MTD, RP2D, AEs	ORR, DCR
BPI-15086	NCT02914990	Phase 1	36	AEs	Cmax, AUC, ORR, PFS
Alflutinib	NCT05364073	Phase 1	170	AEs, ORR	DOR, DCR, PFS, etc.

^a^ EGFR-TKIs, epithelial growth factor receptor tyrosine kinase inhibitors; ORR, overall response rate; MTD, maximum tolerance dose; PFS, progression-free survival; RP2D, recommended phase 2 dose; DOR, duration of response; DLT, dose-limiting toxicities; AEs, adverse events; OS, overall survival; Cmax: peak concentration; AUC, area under the curve; DCR, disease control rate.

## 3 Strategies to address resistance

### 3.1 New-generation EGFR-TKIs

Mobocertinib (TAK-778) is an oral EGFR TKI, primarily targeting patients with NSCLC harboring EGFR exon 20 insertion mutations who exhibit primary resistance to first- and second-generation TKIs. EXCLAIM-2 is a Phase 3 clinical trial aiming to compare the efficacy of mobocertinib versus platinum-based chemotherapy as first-line treatment for EGFR ex20ins + advanced/metastatic NSCLC (Jän et al., 2025). The results showed that the objective response rate (ORR) for the mobocertinib group and the chemotherapy group were 32% and 30%, respectively; the median duration of response (DOR) reached 12.0 months and 8.4 months, respectively. However, the main result of this experiment was not reached, failing to prove the efficacy of mobocertinib over platinum-based chemotherapy.

NCT04036682 (Piotrowska et al., 2025) evaluated the efficacy and safety of zipalertinib (CLN-081) in previously treated patients with NSCLC, typically those with locally advanced or metastatic disease carrying the EGFR ex20ins mutation who had received prior platinum-based chemotherapy. Under the recommended phase 2 dosage, the confirmed ORR was 38%–41%; the median DOR exceeded 10 months, indicating good durability of the efficacy. In patients with brain metastasis, zipalertinib also shows initial intracranial disease control ability. Further, Zipalertinib demonstrated a lower incidence of Grade 3 or higher treatment-related adverse events, representing a significant enhancement in safety profile versus conventional multi-targeted TKIs. The most common grade ≥3 treatment-related adverse events (TRAEs) were anemia, pneumonitis, rash, alanine aminotransferase, and platelet count. In conclusion, zipalertinib showed clinically meaningful efficacy and manageable safety in patients with EGFR exon 20 insertion mutation NSCLC.

To solve the resistance of the third-generation EGFR-TKIs, particularly the resistance to osimertinib caused by the C797S mutation, several new-generation EGFR inhibitors have been developed.

In 2016, EAI045 was discovered as the first new generation of multifunctional inhibitors. Researchers expected it to overcome resistance to third-generation TKIs, but later the development of EAI045 was ultimately discontinued, as investigators discovered that EAI045 was only clinically effective when combined with cetuximab and had harmful side effects on wild-type EGFR, which caused the inconvenience of clinical utilization (Jia et al., 2016; Wang et al., 2017).

JBJ-09-063, formerly known as JBJ-04-125-02, was modified to become the next-generation EGFR allosteric inhibitor, as researchers discovered that the single agent JBJ-04-125-02 was ineffective in preclinical models (To et al., 2022). Ciric et al. validated the efficacy of JBJ-09-063 *in vivo* and *in vitro* and found that single-agent or in combination with TKIs was effective against models of EGFR sensitivity or resistance caused by C797S or T790M mutations (To et al., 2022). The researchers also found that homodimerization or heterodimerization with EGFR or ERBB family members, or L747s mutations, may mediate resistance to JBJ-09-063. Further studies are required for the clinical efficacy of JBJ-09-063, the penetration of JBJ-09-063 into the central nervous system, and the potential for resistance in the future.

BLU-945, an effective fourth-generation TKI, can significantly suppress EGFR-sensitive mutations (exon 19 or L858R), T790M mutations, and C797S mutations in patients with NSCLC (Eno et al., 2022). Results of the SYMPHONY Phase 1/2 Study announced at the American Society of Clinical Oncology (ASCO) conference in 2023 (Elamin et al., 2023). The trial evaluated the safety, tolerability, and clinical efficacy of BLU-945 monotherapy (cohort A, n = 112) or in combination with osimertinib (cohort B, n = 55) in advanced NSCLC patients with EGFR-TKI resistance. After treatment for 15 days, 48% of patients in cohort A had tumor reduction when receiving a dose of ≥400 mg/d, but the persistence of benefit was limited; Nine patients in cohort B achieved a partial response (PR), and tumor shrinkage was seen at a dose ≥300 mg/day. However, BLU-945 showed restrictive toxicity at high doses, and the effective dose of the combination group exceeded the restrictive toxicity dose, resulting in limited clinical usage. Based on this phenomenon, the 42nd Annual J.P. Morgan Healthcare Ombudsman Conference in 2024 proclaimed no further development of BLU-945.

BDTX-1535, a new generation TKI, has a powerful capability of central nervous system penetration and can inhibit more than 50 EGFR mutations, including classical driver mutations, non-classical driver mutations, and acquired resistance mutations, which are expressed in a wide range of NSCLC patient populations (Author anonymous, 2021). Updated its data on efficacy at the latest American Association for Cancer Research (AACR) conference (Wen et al., 2024). 11 patients with resistance to osimertinib were treated with BDTX-1535, and 6 patients achieved PR with an ORR of 55 percent. A Phase 2 trial of BDTX-1535 is underway, and the results are expected to demonstrate the efficacy and safety of BDTX-1535 to NSCLC patients in the first-line or 2/3-line setting. Currently, BDTX-1535 has been approved for Fast Track status by the FDA and is planned to be used for the second-line treatment of NSCLC patients with the EGFR C797S mutation.

JIN-A02 is an entirely novel, oral, fourth-generation TKI that targets C797S and penetrates the blood-brain barrier. Preclinical studies found that JIN-A02 exhibited promising anti-tumor activity in NSCLC patients with EGFR double or triple mutations (exon 19 deletion/T790M mutation or exon 19 deletion/T790M mutation/C797S mutation). Phase 1/2 study of JIN-A02 was announced at the 2024 ASCO meeting (Lim et al., 2024). PR was obtained in 2 patients, and stable disease (SD) in 3 patients. One patient with brain metastasis showed a 28.6% (100 mg/d) reduction in lesion size and improved clinical benefits with increasing dose. Presently, JIN-A02 is still being studied, and it is expected to be the most promising targeted medication in the backline treatment options for advanced NSCLC patients with the C797S and/or T790M mutations.

Enozertinib (BPI-7711) is a novel EGFR TKI with high selectivity and blood-brain barrier permeability. A study explored preclinical and early clinical activity of enozertinib in NSCLC with EGFR 20 exon insertion mutations and atypical mutations (Junttila et al., 2026). Enozertinib has a significant inhibitory effect on multiple EGFR 20ins mutants and non-classical site mutations. In subcutaneously transplanted tumor models, enozertinib induced considerable tumor regression and even achieved complete remission in certain models. Beyond this, the study unexpectedly revealed that enozertinib efficiently crosses the blood-brain barrier, achieving effective therapeutic concentrations within brain tissue. This represents favorable information for patients with EGFR-mutated lung cancer experiencing brain metastases. In terms of safety, AEs associated with enosertinib are comparable to those of other TKIs, with low dose-limiting toxicities, indicating a good therapeutic window.

Fascinatingly, Ahmad et al. proposed a novel class of orthoallosteric bivalent inhibitors. These inhibitors combine the advantages of orthosteric inhibitors (targeting the classical ATP-binding pocket) and allosteric inhibitors (targeting allosteric sites distant from the active site). This combination significantly enhances affinity and selectivity, enabling effective targeting of the C797S mutation (Ahmad and Patel, 2024). A study has proposed a class of ATP-allosteric bivalent inhibitors that simultaneously occupy the ATP pocket and a unique αC-helix-out allosteric channel (Wittlinger et al., 2024). This achieves ultra-high selectivity against EGFR mutants, including L858R/T790M/C797S and L858R/T790M, establishing a novel molecular design paradigm for overcoming resistance to third-generation EGFR-TKIs. It also provides a structural biology foundation for fourth-generation EGFR-TKIs.

FL30 is a novel compound and also an orthoallosteric bivalent inhibitor. Research has found (Romagnoli et al., 2025) that FL30 exhibits potent inhibitory effects on lung cancer cells harboring triple EGFR mutations, effectively overcoming drug resistance caused by T790M and C797S mutations. Compared to conventional inhibitors, FL30 does not directly antagonize the C797S mutation site. Instead, it employs a unique binding and conformational regulation mechanism to render the entire mutated EGFR kinase inactive. In NSCLC models, FL30 effectively inhibits cancer growth and EGFR phosphorylation in EGFR-mutant cells. Current therapeutic strategies increasingly focus on designing TKIs that combine orthosteric binding with allosteric modulation. These findings highlight the potential for further optimization of FL30 and provide new insights for developing drugs targeting other mutable targets.

However, research on this type of inhibitor remains in the preclinical stage, and its efficacy and safety will require repeated validation through standardized, rigorous randomized controlled trials in the future. More fourth-generation EGFR-TKIs are in pre-clinical or clinical trials, and we expect more surprises in the future. Table 3 shows some early clinical trials for fourth-generation EGFR-TKIs.

**TABLE 3:** Some ongoing trials for fourth-generation EGFR-TKIs^a^.

Drug name	NCT number	Enrollment	Primary end points	Secondary end points	Phase
H002	NCT05519293	76	DLT, TEAEs, ORR	Cmax, Tmax, DCR, etc.	Phase 1/2a
TAS-3351	NCT05765734	200	AEs, DLT, ORR	DOR, DCR, PFS, etc.	Phase 1/2
BBT-207	NCT05920135	92	RP2D, AEs, PR, or CR	Cmax, Tmax, TTR, DOR, etc.	Phase 1/2
TRX-221	NCT06186076	115	MTD/RP2D, ORR	Cmax, PFS, AEs, SAEs	Phase1/2
BPI-361175	NCT05393466	30	RP2D, AEs	Tmax, MTD, Cmax, etc.	Phase 1/2
BBT-176	NCT04820023	45	DLT, ORR	Cmax, PFS, DOR, etc.	Phase 1/2
WJ13404	NCT05662670	162	AEs, SAEs, DLT, ORR	Cmax, Tmax, DOR, etc.	Phase 1/2
HS-10375	NCT05435248	354	MTD, ORR	AEs, Cmax, DOR, etc.	Phase 1/2
ES-072	CTR20180074	19	Tmax, RP2D, safety	ORR, DCR	Phase 1
NX-019	NCT05514496	258	TEAE, AEs, SAEs, ORR	PFS, ORR, Cmax, etc.	Phase 1
QLH-11811	NCT05555212	72	AEs, SAEs, ORR	Possible metabolites	Phase 1
TQB-3804	NCT04128085	30	DLT	Cmax, Tmax, PFS, etc.	Phase 1

^a^ EGFR-TKIs, epithelial growth factor receptor tyrosine kinase inhibitors; ORR, overall response rate; MTD, maximum tolerance dose; PFS, progression-free survival; RP2D, recommended phase 2 dose; DOR, duration of response; CR, complete response; DLT, dose-limiting toxicities; PR, partial response; AEs, adverse events; TEAE, treatment emergency adverse event; SAEs, serious adverse events; Cmax: peak concentration; AUC, area under the curve; Tmax, Time to peak drug concentration; TTR, time to response; DCR, disease control rate.

### 3.2 Combination therapy strategies

#### 3.2.1 Dual-target combinations

MET amplification and HER2 overexpression are among the mechanisms mediating acquired resistance to EGFR-TKIs. Dual-targeted combination therapy may be a first-line treatment option for patients with advanced NSCLC with EGFR mutations and MET amplification or HER2 overexpression.

The insight 2 trial (NCT03940703) assessed the clinical efficacy and safety of tepotinib, a MET inhibitor, in conjunction with osimertinib in patients with NSCLC with EGFR-mutated, MET-amplified tumors who had developed resistance after treatment with osimertinib (Wu et al., 2024). In 98 evaluable recipients, the ORR was 50% (n = 49). In the 49 patients who achieved a response, the median PFS was 8.5 months. Of the 24 patients with brain metastases, the intracranial ORR was 29.2%, and 6 received a complete response (CR), and 1 PR. The most common AEs of grade ≥3 are peripheral oedema, decreased appetite, and pneumonitis. This study indicates that osimertinib in combination with MET inhibitors can be a potential treatment option for TKI-resistant patients with EGFR mutation and MET amplification.

Liam et al. assessed the antitumor activity of tepotinib in combination with gefitinib compared with traditional chemotherapy in advanced or metastatic NSCLC patients with MET-altered and EGFR-mutated tumors who had developed resistance after first- or second-line treatment with TKIs (Liam et al., 2023). Among the 19 recipients with MET amplification, the ORR was significantly improved in the combination group than in the chemotherapy group (66.7% vs. 42.9%), and the median DOR was prolonged by 17.1 months (19.9 vs. 2.8 months). The independent review committee (IRC) assessed PFS was 19.3 months in the combination group, compared with only 4.4 months in the chemotherapy group. The proportion of grade ≥3 TRAEs was similar between groups (51.6% vs. 52.2%), and patients were well tolerated.

The FLOWERS trial (NCT05163249) is the first to evaluate the clinical benefit of osimertinib plus sevotinib versus single-agent osimertinib in the first-line treatment of advanced NSCLC patients with EGFR mutation and MET overexpression or amplification (Li A. et al., 2023). MET overexpression will be defined as tissue immunohistochemistry (IHC) 3+ in ≥75% of tumor cells and MET amplification by tissue fluorescent *in situ* hybridization (FISH; MET gene copy number of tumor cells [GCN] ≥5 or MET/CEP7 ratio ≥2 or tissue next-generation sequencing [NGS]-MET CN ≥ 5). At a median follow-up of 8.2 months, the combination group had a considerably higher ORR than the single-agent group (90.5% vs. 60.9%), and patients achieved more long-term alleviation with longer follow-ups. Median PFS was prolonged by 10.3 months in the combination group (19.6 vs. 9.3 months), and the risk of disease progression or death was decreased by 41% versus the single-agent group. TRAEs in both groups were mostly low-grade, and the safety was controllable. More clinical data from this study are still in follow-up, and we expect further surprises to emerge soon.

The TRAEMOS trial evaluated the efficacy and safety of osimertinib in combination with trastuzumab-emtansine in patients with EGFR mutation-positive NSCLC who developed HER2 overexpression after resistance to osimertinib. It was found that ORR after 12 weeks of treatment was 4% (n = 1/27), and mPFS was 2.8 months. The most frequent TRAEs of any grade were fatigue, diarrhea, and nausea, with no deaths. The investigators concluded that while the safety profile of the combination regimen was promising, the efficacy was limited, and further clinical trials were not warranted (Jebbink et al., 2023).

#### 3.2.2 Immunotherapy plus chemotherapy

Lu et al. presented the results of the second interim assessment of the ORIENT-31 study (NCT03802240) (Lu et al., 2023). This trial first demonstrated the efficiency and safety of immune-combination regimens in patients with NSCLC with TKI resistance. The recipients were divided into 3 groups based on the ratio of 1:1:1: chemotherapy alone (pemetrexed plus cisplatin) (control group, n = 160), sintilimab plus IBI305 and chemotherapy (trial group A, n = 158), and sintilimab plus chemotherapy (trial group B, n = 158). The median PFS respectively were 4.3, 7.2, and 5.5 months in the three groups, with the longest PFS in the trial group A. The trial A and B groups had a median OS of 21.1 and 20.5 months, respectively, compared to 19.2 months in the chemotherapy alone group. Subgroup studies revealed that this combination regimen was more beneficial to patients with negative T790M expression. Safety results were similar to the first analysis, with no new safety signals (Lu et al., 2022b).

Jiang et al. demonstrated the efficacy and tolerability of toripalimab plus chemotherapy as a second-line treatment for NSCLC with previous first- or second-generation TKI resistance and EGFR mutations, with no patients (n = 40) having EGFR T790M mutation at the time of biopsy (Jiang T. et al., 2021). The overall ORR and disease control rate (DCR) were 50.0% and 87.5%, respectively. The median PFS and OS, respectively, were 7.0 and 23.5 months. In 90% of patients (n = 36/40), tumor shrinkage was observed. 39 patients experienced any grade TRAEs, and the most common grade 3 or higher TRAEs included leukopenia, neutropenia, anemia, etc. Subgroup analyses showed that patients with programmed cell death 1 ligand 1 (PD-L1) expression positive had significantly longer median PFS and OS (7.6 vs. 5.8 months and NR vs. 21.0 months).

The Checkmate-722 (nivolumab plus chemotherapy) and KEYNOTE-789 (pembrolizumab plus chemotherapy) studies both evaluated the clinical efficacy and safety of immunotherapy in combination with chemotherapy (Mok et al., 2024; Yang et al., 2024). Both studies found that immunotherapy plus chemotherapy did not significantly improve PFS compared with chemotherapy alone, and they did not find new safety events. In a *post hoc* PFS subgroup analysis of the Checkmate-722 study, immunotherapy plus chemotherapy showed a more favorable tendency in patients with tumors harboring sensitizing EGFR mutations or who received only first-line EGFR-TKI treatment. In the KEYNOTE-789 trial, there was a trend toward improved OS in patients with PD-L1 TPS ≥1% for pembrolizumab plus chemotherapy versus placebo plus chemotherapy compared with PD-L1 TPS <1%. But these results do not demonstrate that this combination regimen may serve as the best choice in the future; further researches are needed to confirm it.

#### 3.2.3 Other combination therapies

A phase 3 trial (Park et al., 2024) (NCT03991403) was first conducted in South Korea to assess the clinical efficacy and tolerability of atezolizumab in conjunction with bevacizumab and chemotherapy (paclitaxel plus carboplatin, ABCP) in patients with EGFR-mutated or ALK-mutated NSCLC whose disease had progressed after treatment with TKIs, and 225 patients were randomly assigned to the ABCP group and the PC group (pemetrexed plus cisplatin or carboplatin). The ORR was significantly higher in the ABCP group versus the PC group (69.5% vs. 41.9%). In the ABCP group (n = 151), 1 case achieved CR and 104 cases reached PR. 31 cases in the PC group (n = 74) achieved PR. The median PFS of the 2 groups, respectively, were 8.48 and 5.62 months. The median OS was 20.63 and 20.27 months, respectively. Furthermore, in the brain-metastasis patient population, the median PFS was significantly prolonged in the ABCP group (8.41 vs. 4.40 months), whereas PFS showed no significant difference between the two groups in the no-brain metastasis group. TRAEs of any grade were detected in 96.7% and 75.7% of the two groups, respectively. The ABCP group had a greater incidence of grade 3 or higher TRAEs than the PC group (35.1% vs. 14.9%), which was considered to be related to cytotoxic chemotherapeutic agents. The rate of PFS benefits is higher with increased PD-L1 expression, but the exact population of benefit requires further investigation.

The IMpower 150 study (Socinski et al., 2021), on the other hand, evaluated the efficacy and safety of the ABCP regimen versus the BCP regimen in metastatic non-squamous NSCLC without chemotherapy, to verify whether the ABCP regimen prolongs PFS and OS in patients. In the final analysis, median OS was 4.8 months longer in the ABCP group than in the BCP group (19.5 vs. 14.7), a statistically significant difference. Median PFS was 8.3 and 6.8 months in both groups, respectively. The security characteristics of the ABCP regimen are consistent with previous studies. The above trial results support the ABCP regimen as first-line treatment for patients with metastatic nonsquamous NSCLC, especially in patients harboring sensitive EGFR mutations or in the presence of liver metastases at baseline.

MARIPOSA is a Phase 3, multicenter randomized controlled trial (Cho et al., 2024) to assess the antitumor activity and safety of amivantamab in combination with lazertinib versus osimertinib monotherapy as first-line therapy in advanced NSCLC patients with EGFR mutations. 1074 patients were randomly assigned to 3 groups according to the ratio of 2:2:1, including the amivantamab plus lazertinib group (n = 429), the osimertinib group (n = 429), and the lazertinib group (n = 216). Median PFS was significantly longer in the combination group versus in the osimertinib group (23.7 vs. 16.6 months). Among patients who attained a response (combination group, n = 336, and osimertinib group, n = 314), the median DOR was 258 and 168 months, respectively. Table 4 summarizes some ongoing trials of combinations for EGFR-mutated NSCLC patients with TKI resistance.

**TABLE 4:** A few ongoing trials of combinations for EGFR-mutated NSCLC patients with TKI resistance^a^.

NCT number	Comparison	Primary outcomes	Common adverse events	Phase
NCT05388669	Amivantamab plus lazertinib (subcutaneous group, intravenous group)	mPFS: 6.1 and 4.3 months DCR: 30% and 33%	Infusion-related reactions, venous thromboembolism	Phase 3
NCT02438722	Afatinib plus cetuximab	DCR: 54% mPFS: 5.5 months mOS: 16.8 months	Diarrhea, rash, dry skin, paronychia, erythema	Phase 2
jRCTs 031190066	ABCP	mPFS: 7.4 months mOS: 23.1 months DCR: 55.9%	Hypoalbuminemia	Phase 2
NCT 05329025	QL1706 plus chemotherapy	DCR: 45.0% mPFS: 6.8 months	Decreased appetite, anemia, infusion-related reactions, and pruritus	Phase 2
NCT04965090	Amivantamab plus lazertinib	CSF CTCs decreased: 64%, 32% had improvement in neurologic symptoms	Rash, infusion-related reaction, paronychia, fatigue, etc.	Phase 2
ChiCTR 1900028112	Toripalimab plus anlotinib	DCR: 57.9% mPFS: 2.1 months	Hypothyroidism, fatigue, hypertension, etc.	Phase 2
NCT04245085	ABCP	ORR: 62.5% mPFS: 9.4 months	Immune-related AEs	Phase 2
NCT03706287	Anlotinib plus chemotherapy	mPFS: 5.75 months ORR: 47.4%	Hypertension, decreased platelet count, hypertriglyceridemia	Phase 1b/2
NCT02335944	Capmatinib plus nazartinib	ORR: 45.8%, 26.2%, 37.9%, and 32.4%	Peripheral edema, nausea, diarrhea, maculopapular rash	Phase 1b/2
NCT02143466	Osimertinib plus durvalumab (part A and part B)	ORR: 43% and 82% mDOR: 20.4 and 7.1 months mPFS: 9.0 months (part B)	Diarrhea, nausea, decreased appetite	Phase 1b
NCT04077463	Amivantamab plus lazertinib	ORR: 55% mPFS: 19.5 months	EGFR and MET-related toxicities	Phase 1

^a^ EGFR, epithelial growth factor receptor; NSCLC, non-small cell lung cancer; TKI, tyrosine kinase inhibitor; mPFS, median progression-free survival; DCR, disease control rate; mOS, median overall survival; mDOR, median duration of response; ORR, objective response rate; EGFR, epithelial growth factor receptor; MET, mesenchymal to epithelial transition factor; AEs, adverse events.

### 3.3 Emerging therapies in progress

#### 3.3.1 Bispecific antibodies

Amivantamab (JNJ-6372/JNJ-61186372), a bispecific antibody, targets both EGFR and MET, which bind to the extracellular structures of both EGFR and c-MET, preventing their ligands from binding to EGFR and MET, and inhibiting the downstream signaling pathways of them, as well as recruiting immune cells to kill tumor cells (Cho et al., 2023). On 1 March 2024, the FDA authorized amivantamab in combination with chemotherapy for locally advanced or metastatic NSCLC with EGFR exon 20 insertion mutations (Zhou et al., 2023). The approval was based on the results of the PAPLILLON trial.

In addition, Korea approved lazertinib, a novel third-generation EGFR-TKI, for the treatment of patients with locally advanced or metastatic NSCLC who had previously received EGFR-TKIs therapy and tested positive for the T790M mutation in 2021 (Dhillon, 2021).

A global phase 3 trial (MARIPOSA-2) evaluated the clinical efficacy of amivantamab plus chemotherapy with or without lazertinib in advanced NSCLC patients with EGFR mutations (exon 19 deletions or L858R) after osimertinib resistance (Passaro et al., 2024). 657 recipients were randomly divided into three groups, including the amivantamab plus chemotherapy group, the chemotherapy alone group, and the lazertinib group. Median PFS was 6.3, 4.2, and 8.3 months in the three groups, respectively. Compared with the other two groups, the amivantamab plus chemotherapy and the lazertinib group had a significantly longer PFS and a 52% and 56% lower risk of disease progression or death, respectively. The ORR of the three groups was 64%, 36%, and 63%, respectively. In addition, the median intracranial PFS was highest in the amivantamab plus chemotherapy and lazertinib group at 12.8 months, versus 12.5 and 8.3 months in the other two groups, respectively. Consideration is related to the CNS activity of lazertinib. The most common AE of ≥3 grade was hematological toxicity, with the highest incidence (92% vs. 72% and 48%) in the amivantamab plus chemotherapy and lazertinib group, and the majority of grade 1 or 2 events. In conclusion, the investigators believe that the two trial groups were able to significantly improve PFS in patients with advanced or metastatic NSCLC with EGFR mutations after osimertinib resistance compared with chemotherapy, which could represent a novel treatment strategy in the future.

Amivantamab offers a novel therapeutic alternative for NSCLC patients resistant to third-generation EGFR-TKIs and MET amplification (Yun et al., 2020). Previously, based on the results of the CHRYSALIS Study, the FDA approved amivantamab for second-line therapy in adult NSCLC patients with EGFR ex20ins in May 2021 (Chon et al., 2023). At present, clinical trials of amivantamab in conjunction with other medicines in NSCLC patients with TKI-resistant and EGFR-mutant tumors are ongoing.

Ivonescimab (AK112/SMT112), a humanized IgG1 subtype bispecific antibody, targets human vascular endothelial growth factor-A (VEGF-A) and programmed cell death protein 1 (PD-1). It binds to both VEGF-A and PD-1 and competitively inhibits their interaction with ligands, hence showing remarkable anti-tumor activity (Fang et al., 2024a). In April 2024, ivonescimab was authorized for locally advanced or metastatic non-squamous NSCLC with EGFR mutations and TKI resistance (D and hillon, 2024).

The NCT05184712 trial (Fang et al., 2024a) enrolled 322 patients and evaluated the efficacy and safety of chemotherapy alone (pemetrexed and carboplatin) versus chemotherapy in combination with ivonescimab in advanced NSCLC patients with EGFR mutations who had disease progression following previous treatment with TKIs. The results demonstrated that the median PFS in the combination group was prolonged by 2.26 months (7.06 vs. 4.80 months) and the risk of disease progression or death was reduced by 54% versus the chemotherapy group. The ORR was 50.6% and 35.4% in the two groups, respectively. The rate of ≥3 AEs respectively was 61.5% (n = 99/161) and 49.1% (n = 79/161) in the two groups, which was mainly associated with chemotherapy. The trial indicated that ivonescimab plus chemotherapy, which significantly improved PFS and was well tolerated by patients, could be a potential treatment choice.

BC3448, a bispecific antibody targeting EGFR and CD3, is currently in clinical phase 1 and is used to treat solid tumors (including NSCLC) with high EGFR expression (Guo et al., 2023). Preclinical trials have indicated that BC3448 has promising pharmacodynamic activity in cells or animal models with high EGFR expression, EGFR mutation, or Kirsten rat sarcoma viral oncogene homolog mutation. The results of the following clinical studies are anticipated.

SMET12 is also a bispecific antibody that targets both EGFR and CD3 and induces T cell activation and proliferation by co-binding EGFR-positive tumor cells and CD3-positive T cells, releasing related cytokines to kill tumor cells, and it is expected to overcome the resistance issues of traditional chemotherapy agents, monoclonal antibodies, and TKIs (Liu and Zhu, 2024). Related clinical trials are in progress, including NCT06208033, NCT06208033, and ChiCTR2400079726. Furthermore, AFM24 (NCT05099549), a bispecific antibody targeting EGFR and CD16a, has entered clinical trials.

#### 3.3.2 ADCs

Patritumab deruxtecan is a novel ADC that consists of a human immunoglobulin G1 Mab (pertuzumab) targeting HER3 via a tetrapeptide cleavable linker conjugated to topoisomerase I inhibitor payload (MAAA-1181a) (Coleman et al., 2023).

U31402-A-U102, a global, multicenter phase 1 trial (Janne et al., 2022; Yu et al., 2024) (NCT03260491), assesses the clinical efficacy and safety of patritumab deruxtecan (HER3-DXd, U3-1402) in patients with advanced or metastatic NSCLC with EGFR mutations or acquired resistance to TKIs. The overall ORR was 39%, including 1 CR, 21 PR, and 19 SD. The DOR is 6.9 months. The ORR of 52 patients with brain metastases was 32% (25/52). This study demonstrated the preliminary clinical activity of patritumab deruxtecan.

Similarly, the HERTHENA-Lung 01 trial (Yu et al., 2023) (NCT04619004) evaluated the efficacy and safety of patritumab deruxtecan in NSCLC patients with EGFR-mutated tumors, and 225 participants received 5.6 mg/kg of HER3-DXd every 3 weeks. A median DOR of 6.4 months, a median PFS of 5.5 months, and a median OS of 11.9 months were obtained from the efficacy assessment. The investigators observed similar antitumor activity in patients with or without a history of CNS metastases. The most common grade ≥3 TRAE was hematologic toxicities with a manageable safety profile. The trial concluded that patritumab deruxtecan showed promising clinical efficacy and durable remission rates in NSCLC patients with EGFR-mutated who had previously not responded to platinum-based chemotherapy or TKIs. The phase 3 HERTHENA-Lung 03 trial (NCT05338970), which is still under enrollment, evaluated the safety and efficacy of patritumab deruxtecan in patients with metastatic or locally advanced NSCLC who had failed treatment with EGFR-TKIs compared with platinum-based chemotherapy and is expected to be completed in 2026.

HER3 is frequently highly expressed in EGFR-mutant tumors and is associated with TKI resistance (Ou et al., 2024). BL-B01D1 is created by coupling an anti-EGFR IgG1 antibody via a glycine-serine linker with two anti-human HER3 single-chain fragment variables. Compared to most single-target ADC drugs, BL-B01D1 targets both EGFR and HER3, and this dual-target design enables it to more fully inhibit signaling pathways in tumor cells. Besides, BL-B01D1 contains a novel topoisomerase I inhibitor with powerful cytotoxicity that is efficiently released in tumor cells, enhancing anti-tumor activity. Thus, dual-target design and efficient cytotoxicity of BL-B01D1 make it uniquely superior in addressing TKI resistance (Ma et al., 2024). At ASCO 2023, Professor Li Zhang of China presented an oral presentation on the safety and effectiveness of BL-B01D1 in locally advanced or metastatic solid tumors (including EGFR-mutated and EGFR wild-type NSCLC) that have failed to respond to standard therapy. The overall ORR was 45.3% in this phase 1 trial. The ORR was achieved in 63.2% of 38 participants with EGFR-mutated NSCLC, of whom 34 were resistant to prior TKI therapy. The phase 3 trial (NCT06382116) is ongoing to compare the efficacy and safety of platinum-based chemotherapy (pemetrexed plus cisplatin or carboplatin) with BL-B01D1 in patients with locally advanced or metastatic non-squamous NSCLC with EGFR-sensitive mutations after failure of EGFR-TKIs therapy. Results of the trial are expected in May 2026.

HLX42, a novel ADC candidate targeting EGFR, consists of a highly specific humanized EGFR IgG1 antibody linked to a novel small-molecule inhibitor of DeoxyriboNucleic Acid topoisomerase I via a cleavable linker, with both the precise targeting of the antibody and the powerful tumor cell-killing effect of cytotoxic drugs, which has become a hotspot for research (Shan et al., 2023). Currently, HLX42 is granted FDA fast-track designation for the treatment of advanced or metastatic NSCLC patients with EGFR-mutated that have progressed after treatment with third-generation TKIs. In a prior phase 1 trial (NCT06210815), HLX42 showed promising anti-tumor activity in an NSCLC tumor model refractory to therapy with osimertinib or cetuximab and has been approved by the National Medical Products Administration and the FDA for the treatment of advanced or metastatic solid malignancies (Shan et al., 2023).

Sacituzumab tirumotecan (SKB264/SAC-TMT/Mk-2870) is an anti-tumor-associated calcium signal transducer 2 (Trop2) medication developed individually in China. Trop2 is a transmembrane glycoprotein that is expressed at approximately 90% in NSCLC and has emerged as an ideal target for the therapy of NSCLC (Omori et al., 2022). SKB264 consists of a highly expressed Trop2 antigen connected by a cleavable linker and a novel topoisomerase I inhibitor, with a high drug-antibody ratio of 7.4 (Liu et al., 2023). SKB264 binds to Trop2 antigen and lyses under the action of the lysosome, releasing the small-molecule cytotoxic drug T030, which can precisely destroy tumor cells. Moreover, T030 can also penetrate the cell membrane to exert a bystander effect and has the same devastating effect on cancer cells with low or no Trop2 expression (Ch et al., 2023). The results of part 2 of the OptiTROP-Lung03 trial (NCT05631262, CTR20222948) were reported at the annual AACR convention in 2024. In a population of EGFR-mutated patients resistant to previously targeted therapies, the ORR was 60%, the median PFS was 11.5 months, and the median OS was 22.7 months, with the overall safety profile of patients manageable (Fang et al., 2024b). In August 2024, sacituzumab tirumotecan received Center for Drug Evaluation Priority Review status for its intended application in adult patients with locally advanced or metastatic EGFR-mutated NSCLC who have not responded to TKIs and platinum-containing chemotherapy.

Datopotamab deruxtecan (Dato-DXd) is an ADC targeting Trop2. The ORCHARD study (Yu et al., 2021) evaluated the efficacy and safety of osimertinib in combination with Dato-DXd in patients with advanced NSCLC harboring EGFR mutations after progression on first-line therapy with osimertinib. In NSCLC patients with EGFR mutations after osimertinib-resistance, osimertinib plus Dato-DXd showed favorable antitumor activity with an ORR of 36%–43%. The median PFS after the combination of the two agents was 11.7 (6 mg/kg) and 9.5 (4 mg/kg) months, respectively. The safety of the combination therapy was in accordance with the known safety profiles of the medications, and no new safety issues were identified. The results provide an essential reference for further study of the clinical efficacy of osimertinib with Dato-DXd in EGFR-mutated NSCLC.

In addition, two phase 3 trials (NCT05870319 and NCT06305754) are ongoing to test the efficacy and safety of sacituzumab tirumotecan monotherapy or combination with platinum-containing chemotherapy in patients with EGFR-mutated non-squamous NSCLC who have failed treatment with EGFR-TKIs. These findings are expected to accelerate the approval and marketing of sacituzumab tirumotecan, benefiting more advanced NSCLC patients with TKI resistance. Table 5 shows some ongoing studies of novel drugs for EGFR-mutated NSCLC patients with TKI resistance.

**TABLE 5:** Several ongoing studies of novel drugs for EGFR-mutated NSCLC patients with TKI resistance^a^.

Drug name	Target	NCT number	Enrollment	Primary end points	Secondary end points	Phase
MRG003	EGFR	NCT04838548	90	ORR	PFS, DOR, TTR, etc.	Phase 2
REGN5093	MET	NCT04982224	237	DLT, TEAEs, SAEs, etc.	DOR, DCR, TTR, etc.	Phase 1/2
DB-1310	HER3	NCT05785741	287	DLT, TEAEs, SAEs, etc.	Cmax, Tmax, AUC	Phase 1/2
HLX42	EGFR	NCT06210815	42	DLT, MTD	ORR, DOR, PFS, etc.	Phase 1
BB-1705	EGFR	NCT05217693	288	AEs, SAEs, DLT, MTD	Cmax, ORR, PFS, DOR	Phase 1
AZD9592	EGFRx c-Met	NCT05647122	162	AEs, SAEs, DLT, ORR	DOR, DCR, PFS, etc.	Phase 1
YL202	HER3	NCT05653752	80	DLT, AEs	Cmax, ORR, DCR, etc.	Phase 1
SHR-A2009	HER3	NCT05394818	19	TTP, RP2D, AEs or SAEs	Tmax, Cmax, ORR, etc.	Phase 1

^a^ EGFR, epithelial growth factor receptor; NSCLC, non-small cell lung cancer; TKI, tyrosine kinase inhibitor; ORR, overall response rate; MTD, maximum tolerance dose; PFS, progression free survival; RP2D, recommended phase 2 dose; DOR, duration of response; DLT, dose-limiting toxicities; AEs, adverse events; TEAE, treatment emergency adverse event; SAEs, serious adverse events; Cmax: peak concentration; AUC, area under the curve; Tmax, Time to peak drug concentration; TTR, time to response; DCR, disease control rate; TTP, time to progression.

#### 3.3.3 Novel peptides drugs

A study (Kaewjanthong et al., 2022) reported a novel strategy that inhibits lung cancer growth not by directly targeting EGFR kinase activity, but by disrupting EGFR downstream signaling through cell-penetrating peptides containing polyproline structures. In EGFR-dependent lung cancer cell lines (such as HCC827 or H3255 harboring EGFR mutations), treatment with this peptide significantly suppressed cell proliferation and induced cell cycle arrest. It differs from traditional kinase inhibitors that directly target ATP pockets, so it may still be effective against some kinase inhibitor-resistant tumors. Although the study is still in its early stages, it also indicates that this is a new research direction.

Cilengitide is a highly selective integrin-targeted cyclopeptide inhibitor. Integrins are cell surface adhesion receptors that connect the extracellular matrix to the cytoskeleton and can activate intracellular signaling pathways. These pathways frequently interact with the EGFR signaling pathway, serving as a key alternative pathway leading to TKI resistance. A study (Ka et al., 2025) has explored the use of cilengitide in combination with traditional EGFR-TKI to overcome the resistance of lung cancer cells to afatinib. The results showed that while both drugs demonstrated limited efficacy when used alone, their combined administration significantly reduced the viability of afatinib-resistant cells and markedly increased apoptosis rates, indicating a synergistic effect between the two agents. This synergistic effect may be related to the simultaneous inhibition of both the EGFR and integrin pathways. This combination strategy represents a novel potential option for patients with EGFR-mutated lung cancer who have failed afatinib therapy and exhibit activated integrin signaling.

#### 3.3.4 PROTACs

Proteolysis targeting chimera (PROTACs) have emerged as a novel technology capable of recruiting target proteins to E3 ubiquitin ligases via the ubiquitin-proteasome system, thereby inducing their ubiquitination and degradation. This mechanism does not rely on sustained inhibition of kinase activity, thus offering potential to overcome resistance induced by various mutations, including C797S (Wang et al., 2024).

HBE-843 is a new, efficient, highly selective EGFR-targeted PROTAC. Some researchers explored HBE-843 with the aim of providing new therapeutic strategies for patients with EGFR-mutated NSCLC who have developed resistance to existing TKIs (Baig et al., 2026). HBE-843 is a typical PROTAC molecule comprising three components: an EGFR-binding ligand, a linker, and an E3 ligase ligand. It exhibits potent EGFR degradation activity that effectively degrades EGFR protein even in cells harboring triple EGFR mutations. HBE-843 markedly suppressed cell proliferation in NSCLC cell lines carrying various EGFR mutations, including both sensitive and resistant mutations. The animal experiment revealed that the HBE-843 group demonstrated notable tumor growth inhibition compared to the control group. Because HBE-843 prefers degradation of mutation-positive EGFR over wild-type EGFR, it produces significantly fewer adverse reactions than traditional EGFR-TKIs.

Qu et al. designed a CRBN (Cereblon) E3 ligase-based PROTAC (Qu et al., 2021). They observed that CRBN-PROATAC not only degrades EGFR protein via the classical ubiquitin-proteasome pathway but also simultaneously activates the autophagy/lysosomal pathway, thereby achieving efficient clearance of the EGFR L858R + T790M-mutated protein. This highly efficient degradation capacity also prominently suppressed the proliferation of drug-resistant cells and induced apoptosis. Currently, most PROTACs rely on the ubiquitin-proteasome system to degrade target proteins. Therefore, researchers further investigated why CRBN-PROTAC activates autophagy. They considered that this might be related to EGFR protein aggregation, CRBN’s potential involvement in certain autophagy regulatory processes, and the diversity of PROTAC-induced EGFR ubiquitination signaling pathways.

Amiloride is a clinically widely used Potassium-saving diuretic. Researchers studied whether combining amiloride with EGFR-PROTACs or traditional EGFR-TKIs could overcome treatment resistance in NSCLC (Qu et al., 2025). They found that amiloride exhibits synergistic effects with EGFR-TKIs/PROTACs, effectively reducing the viability of resistance cells and inducing apoptosis. The mechanism for overcoming resistance is likely related to either amiloride inhibiting protective autophagy or enhancing the degradation efficiency of PROTACs.

#### 3.3.5 Natural product-based inhibitors

Narciclasine is an alkaloid extracted from plants of the Amaryllidaceae family, such as narcissus, exhibiting antitumor and anti-inflammatory activity (Fürst, 2016). Previous studies revealed that it regulates Rho GTPases and autophagy pathways. Therefore, researchers hypothesize that combining narciclasine with TKIs may overcome resistance by modulating these resistance-related pathways (Sung et al., 2025). The results revealed that narciclasine effectively suppressed the proliferation of resistant cells. More importantly, when combined with EGFR-TKI, narciclasine demonstrated a synergistic effect, dramatically reducing cell viability. Tumor cells survive under drug stress by enhancing autophagy, while narciclasine effectively blocks autophagy flux. Moreover, narciclasine can also regulate RhoA pathways, making resistant cells unable to maintain their aggressive phenotype.

Berberine is an isoquinoline alkaloid extracted from traditional Chinese medicines such as Coptis chinensis (Xiong et al., 2022). It exhibits antitumor activity and regulates cellular autophagy and apoptosis. Chen et al. (Chen et al., 2021) identified that the combination of berberine and icotinib exerted synergistic inhibitory effects on NSCLC cells, achieving effective elimination of tumor cells by simultaneously inducing autophagic cell death and apoptosis. Additionally, they tested the efficacy of combination therapy on TKI-resistant cells, such as H1975 cells harboring the T790M mutation. Since berberine’s mechanism of action does not depend on EGFR kinase activity, it may still be effective against resistant cells. This means that Berberine is expected to become a sensitizer for icotinib, used to treat or prevent TKI resistance. He et al. (He et al., 2022) found that curcumin restores miR-142-5p expression through epigenetic mechanisms. Subsequently, miR-142-5p suppresses autophagy in lung cancer cells by targeting Ulk1, thereby enhancing the sensitivity of NSCLC cells to crizotinib.

**FIGURE 2:**
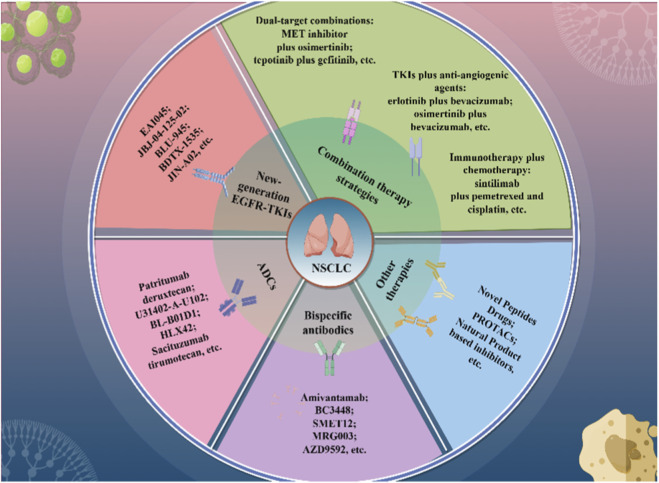
The Emerging strategies for overcoming EGFR-TKI resistance.

Natural product inhibitors possess multi-target advantages, precisely covering resistance pathways such as autophagy, EMT, bypass mechanisms, metabolism, and ferroptosis. They can also enhance sensitivity to TKIs, making them a promising research direction for overcoming TKI resistance. Emerging strategies for overcoming EGFR-TKI resistance are shown in Figure 2.

## 4 Conclusion and future perspectives

Currently, compared to conventional chemotherapy, EGFR-TKIs provide sustained hope for survival in patients with EGFR-mutated non-small cell lung cancer, particularly adenocarcinoma. However, most patients still cannot avoid the problem of resistance to TKIs. Although studies on the mechanism of TKI resistance are gradually improving, the mechanism of resistance in most patients is multifactorial and not fully understood. To identify the mechanisms of TKI resistance, it is necessary to develop novel technologies for detection and improve relevant biomarkers. Multiple therapeutic strategies are available for NSCLC patients with TKI-resistant and EGFR-mutated tumors, but most of them are ineffective and unsustainable. In the era of precision therapy, individualized therapy is particularly crucial. Monoclonal antibodies, ADCs, bispecific antibodies, and combination therapy strategies offer promising treatment options for NSCLC patients with osimertinib-resistant and EGFR mutations. We anticipate that more precision therapeutic approaches and combination regimens, which are currently in the preclinical and clinical trial stages, will tackle the issue of resistance and increase the life expectancy for NSCLC patients harboring EGFR mutations after TKI resistance.

Furthermore, in the first-line treatment of NSCLC patients with EGFR mutations following TKI resistance, there may be notable differences in the mechanisms of resistance between osimertinib monotherapy and combination regimens. These differences primarily arise from the possibility that combination therapy may delay or reverse resistance through multi-target inhibition or synergistic effects, but it may also cause new resistance issues through other pathways or mechanisms. We discovered from the literature research that the C797S mutation, associated gene amplification, bypass signaling pathway activation, histological transformation, tumor immune microenvironment modifications, and other factors were the key causes of the osimertinib resistance mechanism. New resistance issues may emerge, even though the overall resistance incidence is limited with combination therapy strategies. Considering that the mechanisms of resistance for combination therapies are not delineated, we outline several plausible mechanisms of resistance (Bray et al., 2024): Resistance associated with MET amplification. According to the TATTON study, osimertinib combined with MET inhibitors (like savolitinib) effectively overcomes resistance mediated by MET amplification. However, secondary mutations or bypass activation of the MET pathway (like the D1228N mutation) may still result in the emergence of new resistance mechanisms during long-term therapy (Fu et al., 2022). Resistance linked to combined chemotherapy. The FLAURA2 study demonstrated that osimertinib in conjunction with chemotherapy markedly enhanced patient survival; however, post-treatment resistance mechanisms may involve genomic instability of tumor cells, immune cell infiltration patterns, and aberrant activation of DNA repair pathways (Borgeaud et al., 2024). Tumor microenvironment-associated resistance. By altering the immune microenvironment, combination treatment approaches may cause tumor cells to develop resistance issues through immune escape mechanisms (Mok et al., 2017). Bispecific antibody-related resistance. MARIPOSA studies have demonstrated that amivantamab can significantly reduce tumor growth by targeting both the EGFR and MET pathways. However, resistance mechanisms can also involve bypassing signaling pathways, such as the IGF1R and AXL pathways, or activating other targets. Finally, as combination therapy tactics continue to develop, so does research into resistance mechanisms. Summarising the variations in resistance mechanisms between combination treatments and osimertinib alone will help to enhance clinical practice and research in the future, as well as provide an innovative strategy for precision therapy.

## Glossary

EGFR-TKIs: epithelial growth factor receptor tyrosine kinase inhibitors
NSCLC: non-small cell lung cancer
ADC: antibody-drug conjugate
PFS: progression-free survival
ATP: adenosine triphosphate
FDA: food and drug administration
CNS: central nervous system
OS: overall survival
AEs: adverse events
HER: human epidermal growth factor receptor
MET: mesenchymal to epithelial transition factor
RET: rearranged during transfection
BRAF: V-RAF murine sarcoma viral oncogene homolog B1
ALK: anaplastic lymphoma kinase
FGFR: fibroblast growth factor receptor
FGF: fibroblast growth factor
SCLC: small cell lung cancer
TP53: tumor protein p53
JAK/STAT: Janus kinase/signal transducer and activator of transcription
RAS/MAPK/ERK: Ras/mitogen-activated protein kinase/Extracellular regulated protein kinases
PI3K/AKT/mTOR: Phosphatidylinositol-3-kinase/Akt/Mammalian target of the rapamycin
EMT: epithelial-mesenchymal transition
TAM: tumor-associated macrophages
GLMP: glycosylated lysosomal membrane protein
RhoA: Ras Homologous Gene Family Protein A
IGF1R: Insulin-like growth factor 1 receptor
ASCO: American society of clinical oncology
PR: partial response
ORR: objective response rate
SD: stable disease
CR: complete response
DOR: duration of response
DCR: disease control rate
TRAE: Treatment-related adverse event
AACR: American association for cancer research
IRC: Independent review committee
PD-L1: Programmed cell death 1 ligand 1
PD-1: programmed cell death protein 1
VEGF-A: vascular endothelial growth factor-A
Trop2: Tumor-associated calcium signal transducer 2
PROTACs: proteolysis targeting chimera
